# Mechanisms and Contextual Factors Affecting the Implementation of Animal Health Surveillance in Tanzania: A Process Evaluation

**DOI:** 10.3389/fvets.2021.790035

**Published:** 2022-01-13

**Authors:** Janeth George, Barbara Häsler, Erick V. G. Komba, Mark Rweyemamu, Sharadhuli I. Kimera, James E. D. Mlangwa

**Affiliations:** ^1^Department of Veterinary Medicine and Public Health, Sokoine University of Agriculture, Morogoro, Tanzania; ^2^SACIDS Foundation for One Health, Sokoine University of Agriculture, Morogoro, Tanzania; ^3^Department of Pathobiology and Population Sciences, Veterinary Epidemiology, Economics, and Public Health Group, Royal Veterinary College, London, United Kingdom

**Keywords:** process evaluation, animal health, surveillance, Tanzania, contextual factors

## Abstract

A strong animal health surveillance system is an essential determinant of the health of animal and human population. To ensure its functionality and performance, it needs to be evaluated regularly. Therefore, a process evaluation was conducted in this study to assess animal health surveillance processes, mechanisms and the contextual factors which facilitate or hinder uptake, implementation and sustainability of the system in Tanzania. A mixed-method study design was used to evaluate the national animal health surveillance system guided by a framework for process evaluation of complex interventions developed by Moore and others. The system was assessed against standard guidelines and procedures using the following attributes: fidelity, adherence, exposure, satisfaction, participation rate, recruitment and context. Quantitative and qualitative data were collected using a cross-sectional survey, key informant interviews, document review, site visits and non-participant observation. Data from questionnaires were downloaded, cleaned and analyzed in Microsoft™ Excel. Qualitative data were analyzed following deductive thematic and content analysis methods. Fidelity attribute showed that case identification is mainly based on clinical signs due to limited laboratory services for confirmation. Data collection was not well-coordinated and there were multiple disparate reporting channels. Adherence in terms of the proportion of reports submitted per month was only 61% of the target. District-level animal health officials spent an average of 60% of their weekly time on surveillance-related activities, but only 12% of them were satisfied with the surveillance system. Their dissatisfaction was caused by large area coverage with little to no facilitation, poor communication, and lack of a supporting system. The cost of surveillance data was found to be 1.4 times higher than the annual surveillance budget. The timeliness of the system ranged between 0 and 153 days from the observation date (median = 2 days, mean = 6 days). The study pointed out some deviations in animal health surveillance processes from the standard guidelines and their implication on the system's performance. The system could be improved by developing a user-friendly unified reporting system, the active involvement of subnational level animal health officials, optimization of data sources and an increase in the horizon of the financing mechanism.

## Introduction

In the last three decades, there has been increased attention on strengthening health surveillance systems in both animals and humans due to increased threats on emergence and re-emergence of infectious diseases and bioterrorism amplified by cross-border activities among countries, animal movements and livestock trades. A robust animal health surveillance system is a determining factor of the health of animal and human populations. It is used to predict public health risks (1), provide early warning for natural hazards and bioterrorism (2), and enable the disease-free movement of animals and animal-derived products (3). The information and outputs of the surveillance programmes help to set priorities and guide effective disease prevention and control strategies (4). The ability of the system to meet its surveillance objectives is determined by how the data are collected, analyzed and used in solving animal health-related problems. Therefore, to ensure that the systems provide timely and reliable information for planning and decision making and that resources are used efficiently, surveillance systems need to be evaluated regularly (5). The purpose of an evaluation is to assess the functionality, performance and efficiency of a system and to generate recommendations for improvement. Once implemented, they will help to improve the surveillance information provided and thereby help improve service provision and delivery. In early warning surveillance, it is necessary to evaluate the relevance of the selected events, how the system can detect and report them, and how the system can respond to them (6). Evaluation of surveillance programmes is also essential to ensure that limited resources are effectively used to provide the evidence required for protecting animal health (7).

Several evaluation methods have been developed for health surveillance systems, but most of them are from the public health field, while few are from animal health (7, 8). Common evaluation methods mainly apply quantitative approaches such as simulation models, measuring the proportion of cases reported and comparing one system with another; only few use qualitative approaches (7). Furthermore, evaluation is commonly based on the attributes of the systems where the most frequently assessed performance attributes are sensitivity, timeliness and data quality (7). Process evaluation, a predominantly qualitative approach, explains how and why decisions are made, and activities undertaken and assess the reasons for the successful and unsuccessful performance of the programme (9). The primary aim of process evaluation is to determine whether programme activities have been implemented as intended and where they have resulted in certain outputs. By understanding of the processes underpinning the programme's implementation using standardized variables, it will be possible to determine the link between performance, outcomes and factors influencing the implementation (10). It helps to make a distinction between implementation failure and theory failure.

Tanzania's animal health surveillance system has been evaluated using various tools, namely the OIE Performance of Veterinary Services (PVS) evaluation in 2008 and 2016, PVS Gap analysis in 2009 and the FAO Surveillance Evaluation Tool (SET) in 2017. The system was also subjected to a Joint External Evaluation (JEE) in 2016. The second PVS evaluation of 2016 pointed out technical strengths and weaknesses of the surveillance, among other components. It showed that the technical authorities and capacities had not changed since the 2008 evaluation. The underreporting was still high, and more than 90% of the reports were based on clinical signs without laboratory confirmation. Further, there was a limited number of veterinary paraprofessionals, inadequate in-service trainings on surveillance and disease control, insufficient funding and an unclear communication chain (11). The JEE 2016 results were consistent with the second PVS evaluation of the same year (12). The SET 2017 report highlighted strengths in the analytical aspects of laboratory, epidemiology workforce management, training, and internal communication. It also pointed out areas that needed improvement, including unclear roles and responsibilities of partners in the surveillance system, limited supervision, partial harmonization of surveillance activities at the field level, low inter-sectoral collaboration and limited integration between laboratories and the wider surveillance system (13). All these evaluation tools are voluntary, applied by countries upon request and commonly used for self-assessment; overall they aim at identifying critical gaps, strengths and weaknesses in the systemThe PVS and JEE tools focus on the entire veterinary and health service delivery system, of which animal health surveillance is just one of the components. The SET is exclusively dedicated to the assessment of animal health surveillance systems. The tool is relatively new since it was piloted for the first time in Tanzania in 2017 before being adopted by other countries.

Unlike the previous evaluation tools which focused on technical and resource capacities and capabilities using pre-defined set of indicators and scoring system, this study aimed to evaluate surveillance processes, mechanisms and the contextual factors which facilitate or hinder uptake, implementation and sustainability of the system.

Using a process evaluation approach, the objectives were to:

1. Evaluate whether animal health surveillance in Tanzania is implemented as per national and international guidelines.
2. Understand what and how contextual factors influence the implementation of animal health surveillance activities.
3. Explore how implementation processes are linked to the surveillance outcomes.

This study identifies factors related to the successful implementation of animal health surveillance in Tanzania. The evaluation results may provide more insights on intervention areas for improving the surveillance system.

## Materials and Methods

### Study Setting: Tanzania Animal Health Surveillance System

**Figure 1:**
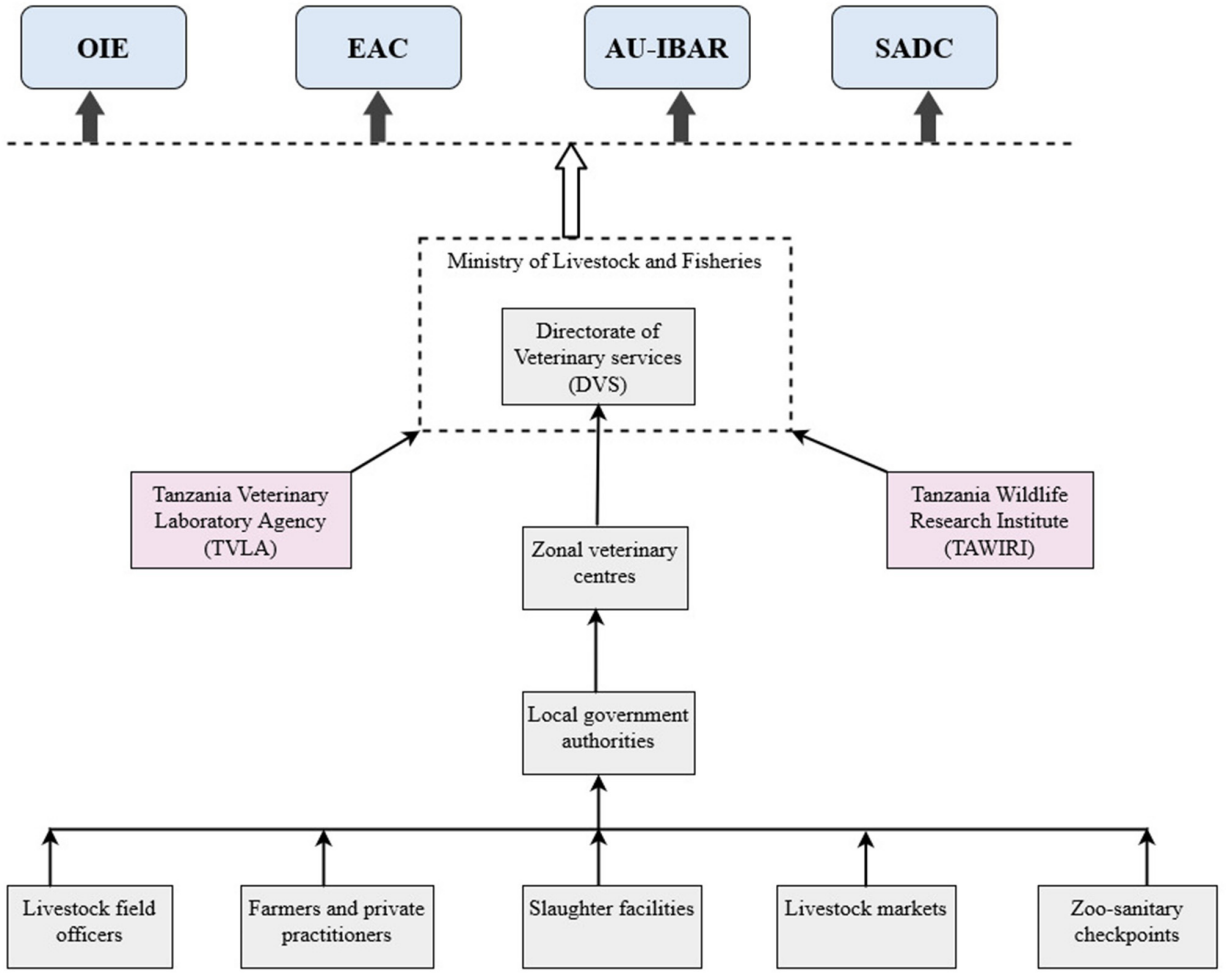
Tanzania's animal health surveillance reporting structure.

The study involved the national animal health surveillance system in Tanzania, coordinated by the Ministry of Livestock and Fisheries (MoLF) through the epidemiology unit in the Directorate of Veterinary Services (DVS). It is a multi-objective system focused on understanding the disease distribution, the introduction of new strains, risks of disease introduction, and vaccination efficiency. The system covers both domestic and wildlife animals. Figure 1 illustrates Tanzania's animal health surveillance reporting structure involving local government authorities, zonal veterinary centres, Tanzania veterinary laboratory agency (TVLA), Tanzania Wildlife research Institute (TAWIRI) and DVS. The primary providers of surveillance information are farmers at the farm level, livestock field officers (LFOs), and district veterinary officers (DVOs) who collect information from veterinary facilities in the areas of jurisdiction. At the zoo-sanitary checkpoints, inspectors are the point of capture, while in wildlife, data flow starts from the park warden. TVLA tests biological samples from zonal veterinary centres (ZVCs) and private clients while TAWIRI tests samples on few selected diseases from wildlife and livestock in the interfaces on annual basis or whenever there is an outbreak. Currently, the two institutions only report notifiable diseases to DVS. The generated reports are shared with various stakeholders, including ministries and international organizations such as World Organization for Animal Health (OIE), East African Community (EAC), African Union (AU), Southern African Development Community (SADC), and Food and Agriculture Organization of the United Nations (FAO).

Surveillance activities include the mandatory reporting of notifiable diseases as per the Animal Disease Act of 2003 and international bodies such as OIE and FAO, ante-mortem and post-mortem inspection reports from slaughter facilities, visual inspection reports from animal congregational areas such as dipping sites, livestock markets and checkpoints, reports on zoonotic diseases and laboratory-based surveillance. George et al. (16) provided a detailed description of each data source including information collected and coverage.

The study involved multi-level data collection. At the national level, seven institutions and six veterinary facilities were conveniently sampled and visited. The institutions included the MoLF, TVLA, TAWIRI, three ZVCs. The visited veterinary facilities included three zoo-sanitary checkpoints, two private poultry farms, and a cattle ranch.

**Figure 2:**
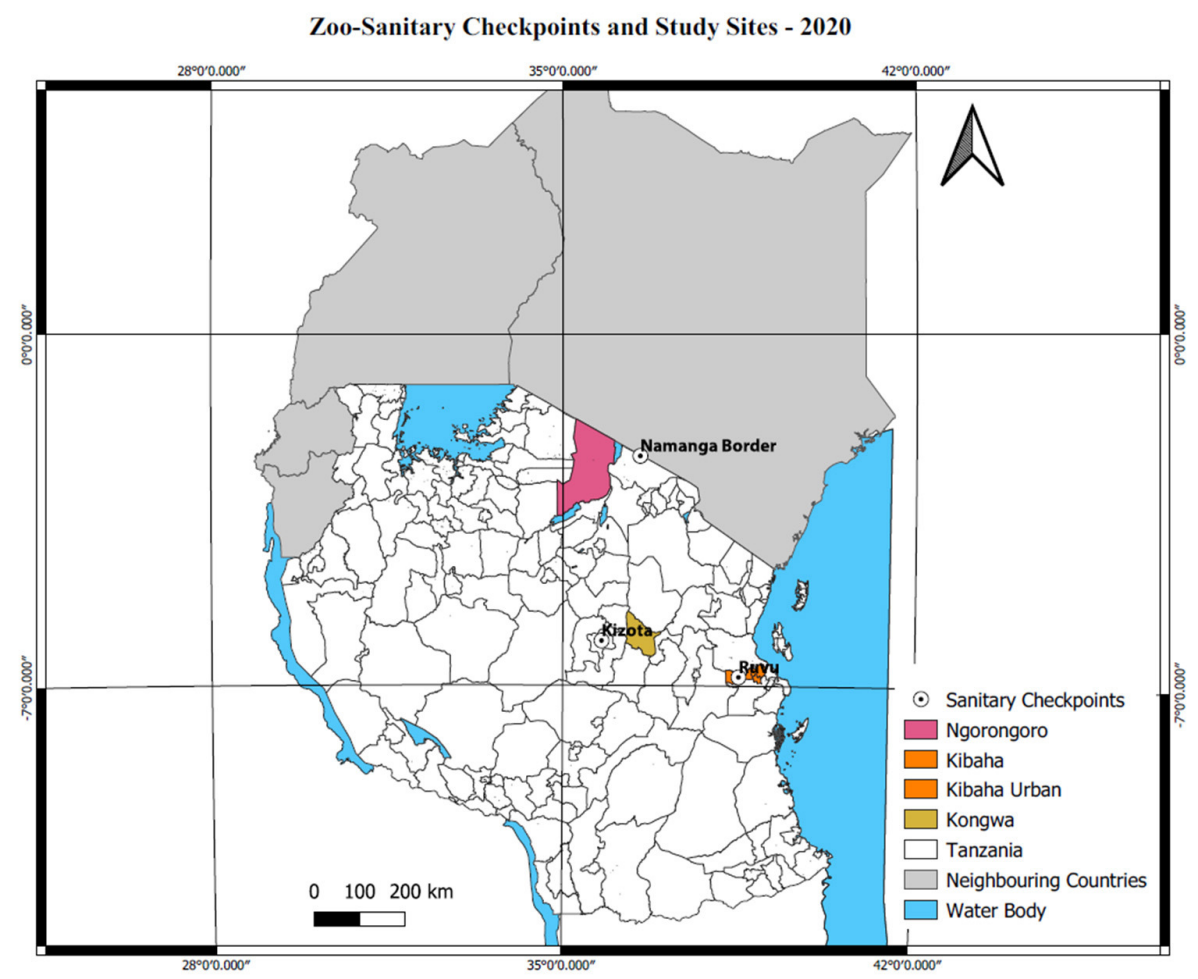
Map of the study districts and zoo-sanitary checkpoints [Personal creation using QGIS version 3.12.3-Bucureşti (14)].

The field investigation was conducted in three districts: Kibaha, Ngorongoro and Kongwa (Figure 2). Selection criteria included the livestock production system, location, cross-border interaction and level of intervention activities in the district. Ngorongoro has high pastoral with a unique human-livestock-wildlife interface. Its closeness to a bordering country (Kenya) was an excellent opportunity to observe cross-border activities related to surveillance and ongoing intervention activities on improvement of human and animal health surveillance systems through mobile technologies. Kibaha is a peri-urban district with mixed livestock production systems, and it was included to observe whether proximity to the city (Dar es Salaam) and has also received some interventions on the improvement of the surveillance system. Kongwa is characterized by high pastoral activities, including national ranches but received interventions in terms of surveillance improvement. From the districts, 10 administrative divisions (hereafter called “wards”) were randomly selected using a random number generator from a list obtained from district economic profile reports and census data. The selection process of the study areas has been explained in detail elsewhere (16).

### Process Evaluation Framework

**Figure 3:**
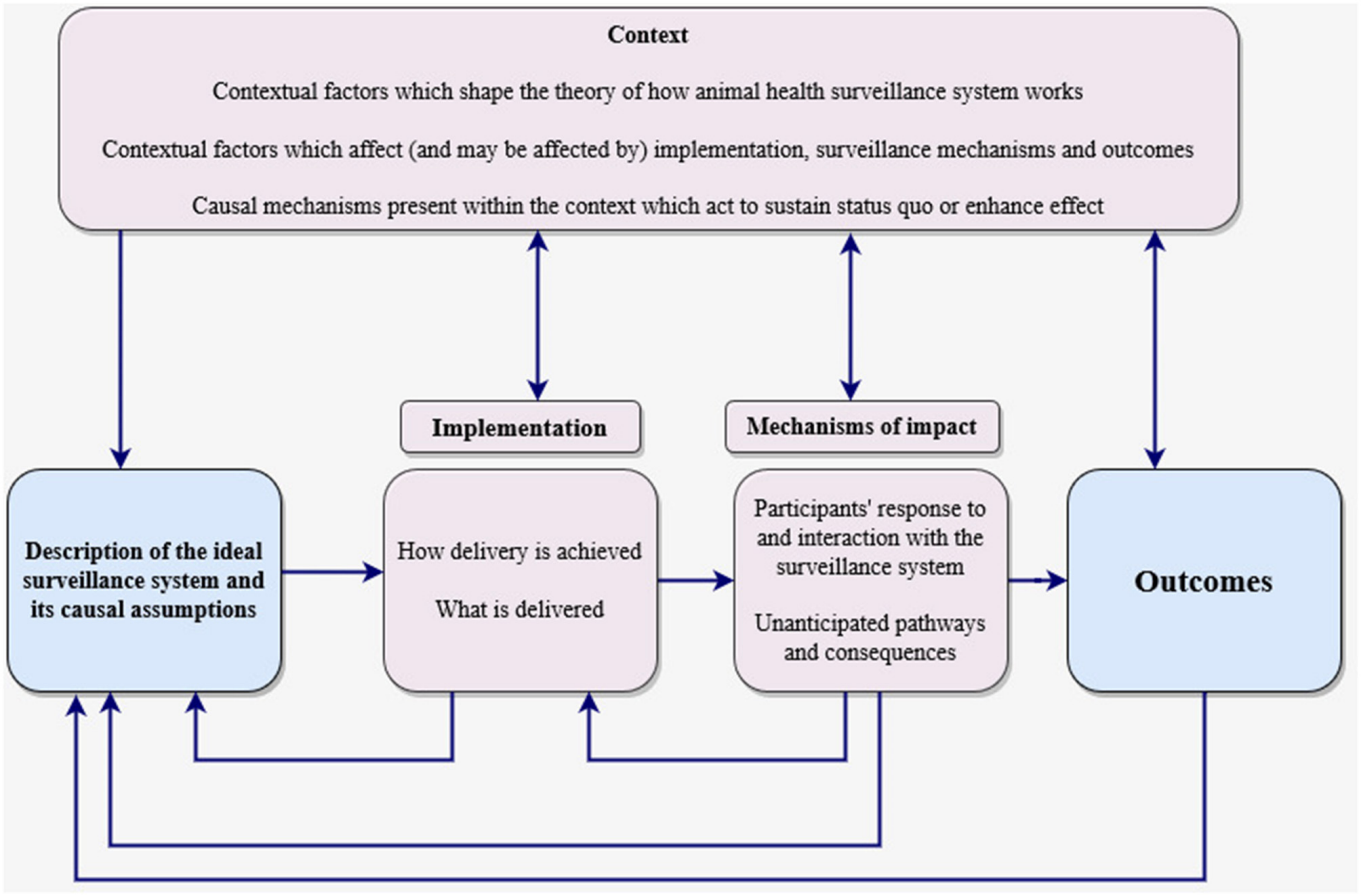
Process evaluation framework adapted from Moore et al. (15) (amour colored boxes are the key components of a process evaluation. Investigation of these components is shaped by a clear description of the surveillance system which also informs interpretation of outcomes).

The study design was guided by a framework for process evaluation of complex interventions developed by Moore et al. (15). Although the framework was originally designed for the evaluation of public health interventions it is highly relevant in other domains as it embrace the systems perspective (17). It has been adapted in this study in order to understand the relationships between animal health surveillance implementation, mechanisms and context in wholesomeness. The framework consists of three main components: implementation, mechanisms of impact, and context. The implementation component captures what is delivered by looking at whether the surveillance components were delivered as intended (fidelity), how they were delivered (completeness/adherence), and the level of exposure (interaction) to the system. Mechanisms of impact explore how the delivered surveillance components produce outcomes by testing and establishing causal pathways using qualitative and quantitative data. Data were collected based on seven process evaluation attributes: fidelity, completeness, exposure, satisfaction, participation rate, recruitment and context. In order to fit the context of this study, the framework has been adapted by replacing intervention with animal health surveillance as illustrated in Figure 3, and defined evaluation attributes as applied in the study (Table 1) while maintaining the key components.

**Table 1:** Process evaluation attributes and data collection instruments.

**No**.	**Attributes and original definitions**	**Application of terms in this study**	**Data collection instrument**	**Participants/information source**
1		Description of the ideal surveillance system and causal assumptions	Key informant interviews	Ministry of Livestock and Fisheries
2	**Fidelity** *Whether the intervention was delivered as intended*	The extent to which the surveillance components have been implemented as per guideline. The following processes were assessed: case identification, reporting, analysis and interpretation, investigation, response and feedback	Questionnaire, record review and non-participant observation	Ministry of Livestock and Fisheries, Livestock field officers, District veterinary officers, Tanzania veterinary laboratory agency, Zonal veterinary centres
3	**Adherence** *Proportion of units delivered against the intended as per protocol*	Number of surveillance reports submitted on time	Key informant interviews and record review	District veterinary officers, Ministry of Livestock and Fisheries
4	**Exposure** *Extent to which the participants were engaged and interact with the system*.	It entailed levels of participation in surveillance activities, communication and feedback mechanisms and training related to surveillance	Questionnaire	District veterinary officers, Livestock field officers,
5	**Satisfaction** *The level of participants' satisfaction with the programme*	Respondents' satisfaction with how the system works. It was measured through ranking using the Likert scale.	Questionnaire	District veterinary officers, Livestock field officers,
6	**Participation** *The proportion of intended target audience that participates in an intervention*	Proportion of respondents who participated in the surveillance system and barriers to their participation. It was measured by the proportion of total working time dedicated to the surveillance activities	Questionnaire	District veterinary officers, Livestock field officers,
7	**Recruitment** *Procedures used to approach and attract participants*	Procedures for recruiting human resource in the systems and sustaining them in the surveillance activities. This was assessed using the education background of animal health professionals, on-job trainings and refresher trainings	Questionnaire and key informant interviews	Ministry of Livestock and Fisheries, Livestock field officers, District veterinary officers, Zonal veterinary centres
8	**Context** *Aspects of the larger social, political, and economic environment that may influence intervention*	Aspects of the larger social, political, and economic environment that may influence animal health surveillance implementation	Questionnaire, key informant interviews and record review	Ministry of Livestock and Fisheries, Livestock field officers, District veterinary officers, Tanzania veterinary laboratory agency, Zonal veterinary centres

**Figure 4:**
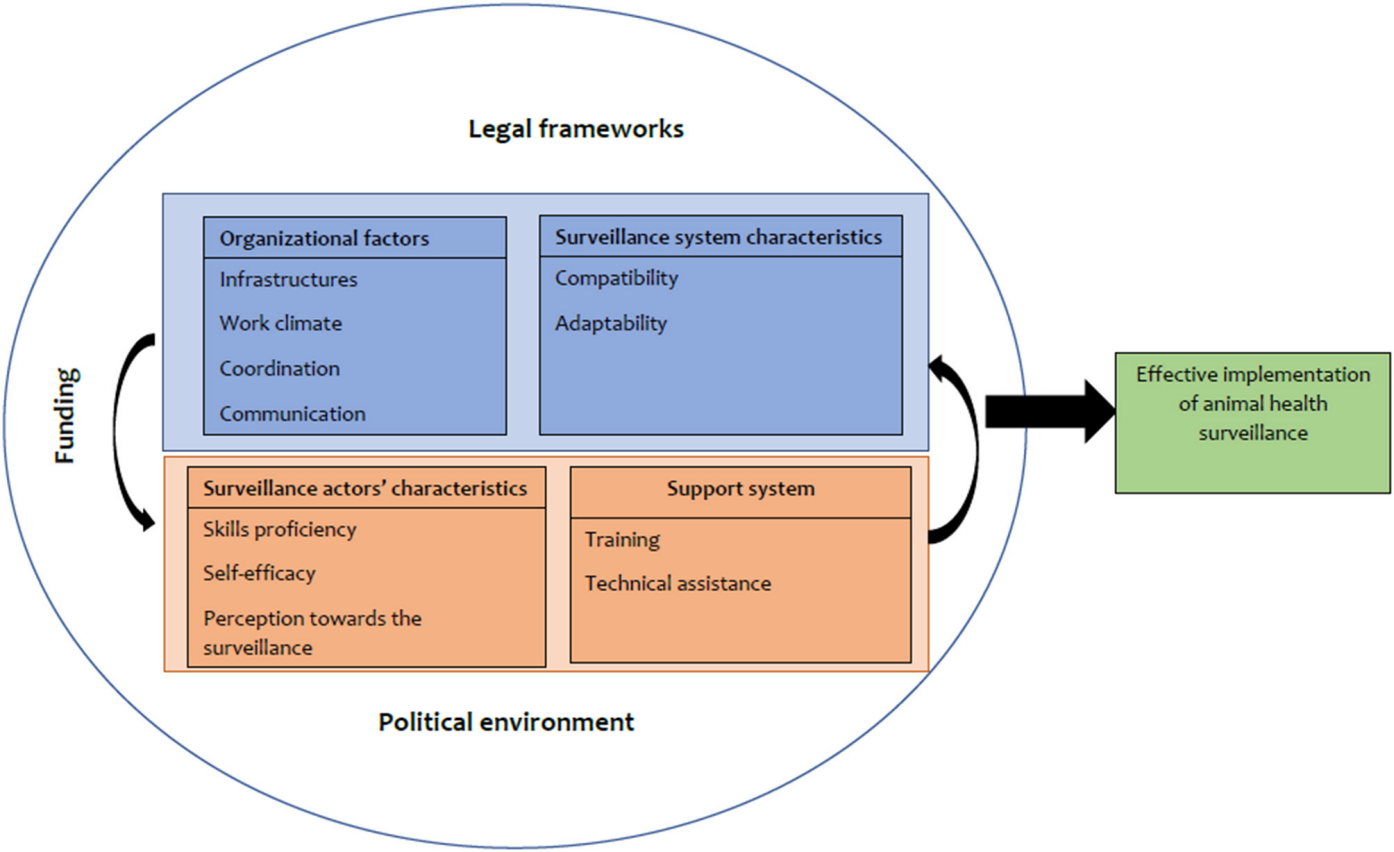
Contextual factors for the implementation of animal health surveillance. The four compartments represent the internal contextual factors of the surveillance system surrounded by external contextual factors in the outer layer which together influence the implementation of the animal health surveillance.

Contextual factors entail all the environmental influences on the surveillance implementation and outcomes. Factors affecting the implementation of animal heath surveillance were grouped into four categories namely: organizational factors, systems characteristics, actors' characteristics and the support system and they are regarded as internal contextual factors of the system (Figure 4). However, the system doesn't operate in isolation and its operations are dependent on the external factors such as legal frameworks, the political environment and funding.

The following outcome variables were included: timeliness, quality of data, usefulness and simplicity, guided by the WHO protocol for evaluation of epidemiological surveillance system (6).

### Selection of Participants

The selection of participants combined random and purposive sampling based on their role in animal health surveillance and designations at their respective institutions. The purposive sampling technique, also called judgment sampling, is the deliberate choice of an informant due to the qualities the informant possesses (18). At the ward level, respondents were LFOs and officers in-charge of the public veterinary facilities. At the district level, DVOs or district livestock officers and livestock officers in-charge of district-level veterinary facilities such as slaughterhouses and livestock markets were interviewed. At the zonal level, respondents were officers in charge of ZVCs and livestock officers at zoo-sanitary checkpoints. At the national level, the interviews were conducted with senior officials responsible for animal health surveillance.

### Data Collection

#### Questionnaire Administration

A structured questionnaire for the quantitative cross-sectional survey was administered to LFOs and DVOs through face-to-face interviews conducted in Swahili language by the first author. The questionnaire included the following closed questions: area coverage, the respondent's role in surveillance, data collection and transmission procedures, communication channels to stakeholders, time dedicated to surveillance activities, conversance with data collection tools, facilitation and costs incurred during implementation of activities, recruitment and trainings. A Likert scale was used to establish participants' satisfaction with the current animal health surveillance system (1-not satisfied at all, 5-highly satisfied). Open-ended questions captured challenges during the implementation of surveillance activities and proposed system improvements. Data were collected using Open Data Kit (ODK).

#### Key Informant Interviews

Semi-structured interviews were conducted with DVOs, in-charge of veterinary facilities, zoo-sanitary checkpoints, and ZVCs. The focus of the interviews was on the data generated and collected from the sources in their designated areas, data management, standard operating procedures (SOPs), and supervision of field staff, the status of the facilities and workforce, internal and external communication and triangulation of some of the data collected through other methods. The interviews were audio-recorded and later transcribed for data analysis.

#### Site Visits to Veterinary Facilities and Non-participant Observation

These methods were used to assess the physical conditions of the veterinary facilities, observe the practices on the sites to compare them with the SOPs established by the animal health authorities and triangulate data obtained through other methods. The visited veterinary facilities included cattle dipping sites, zoo-sanitary checkpoints, slaughter facilities and livestock markets. During the observation, the first author found an inconspicuous spot took notes on the ongoing events and scenes notes on the scenes in real-time. The researcher informed the participants about the reasons for their presence so that they did not change their normal practices and sometimes had post-observation informal interviews with some of them to clarify what was happening and why. The observation period was 1–4 h, and field notes were taken for later analysis. The purpose of the observation was to observe participants' practices and behavior patterns while routine surveillance-related activities went on.

#### Record Review

The reviewed records comprised of monthly reports for 2018/2019 data from Agricultural Routine Data system (ARDS), an official data collection system in the agricultural sector and year 2020 from FAO EMPRES Global Animal Disease Information System (EMPRES-i) which were made available by DVOs and National veterinary epidemiologist respectively. Weekly reports, SOPs documents and animal disease surveillance forms were obtained from MoLF and TVLA while the legal and policy documents were collected from the official government portal (https://www.tanzania.go.tz/), and other websites. The document review was necessary for triangulation and gathering comprehensive information, which were not readily available through other methods (19). Data on the surveillance budget were obtained from the annual ministerial budget speech and the Policy and Planning Division of the MoLF which is responsible for budgeting and planning.

### Data Processing and Analysis

#### Statistical Analysis

Data from questionnaires were downloaded, cleaned and analyzed using Microsoft™ Excel. Descriptive statistics using frequency and simple percentages were obtained to summarize participants' demographics, report submission rate, participation and area coverage.

#### Estimation Methods

Cost of surveillance data included labor charges, data collection, transmission, compilation and analysis at ward, district, zonal and ministry levels. Costs of surveillance data per LFO were calculated by adding the cost of data collection to the cost of transmission with the assumption that every time LFOs do routine visits to the village, they also collect surveillance data. The cost of data collection was calculated by multiplying the total distance traveled per month by the average cost per kilometer. The respondents provided the cost of transmission to the district level during questionnaire administration. The calculation of the costs per kilometer was done using a motorbike as a means of transport as reported by the majority of the LFOs. The average prices of petrol per liter for 2019 were 2375TZS (Ngorongoro), 2256TZS (Kongwa) and 2205TZS (Kibaha) (20). It was estimated that motorbike can use 1 liter per 20 kms based on the informal interviews with random five motorbike riders. The average surveillance data cost per LFO was then multiplied by the number of wards in the country to get the total cost national-wide. There were a total of 3,956 wards in the country (21).


$$\begin{array}{l} Annual\;cost\;of\;surveillance\;data\;per\;LFO=\frac{distance}{year}\\ x\frac{cost}{kilometre}+cost\;of\;data\;transmission \end{array}$$

Where;


$$\begin{array}{l} Total\;distance\;=\;number\;of\;village\;x\;average\;distance\;traveled\\ per\;month \end{array}$$

And


$$\begin{array}{l} Total\;cost\;of\;surveillance\;data\;=\;Tota\;cost\;at\;ward\;level\;+\;cost\\ at\;district\;level\;+\;cost\;at\;zonal\;level\;+\;cost\;at\;ministry\;level \end{array}$$

EMAi-Dataset: Raw data from EMPRES-i database for the year 2020 was downloaded in CSV format and cleaned for analysis of timeliness. Timeliness was measured as difference between observation date and reporting date. Mean, median and rate timeliness also were estimated.

Proportion of monthly reports received at national level was calculated by dividing the total number of monthly reports received from the districts with the total number of monthly reports expected at national level. It should be noted that the reporting is done weekly, and analysis considered all the LGAs which have been reported at least once in that month.

Data for surveillance budget analysis: A 5-year surveillance budget analysis (2015/2016–2019/2020) was conducted by extracting surveillance items in the main budgets. However, it was very difficult to separate surveillance activities from disease prevention and control. Therefore, the analysis included all items mentioned in disease prevention and control in addition to explicitly mentioned surveillance items.

#### Qualitative Data Analysis

Data collected through non-participant observation and site visits were organized and summarized in Microsoft Word. Audio recordings obtained during key informants interviews were transcribed and translated from Swahili into English. Qualitative data were analyzed following deductive thematic analysis. Thematic analysis was guided by Joffe and Yardley (22), whereby data were reviewed, manually coded in MS Word and clustered to establish themes. Documents reviewed were analyzed by using content analysis method (23) which involves open coding of the potential words of interest, creation of categories and abstraction.

## Results

### Demographic Characteristics of the Respondents

At the district level, the questionnaire was administered to 30 livestock field officers (LFOs), two DVOs, and a district livestock officer. Table 2 illustrates the demographic characteristics of the respondents. Thirty-four semi-structured interviews were conducted with designated officials at various levels. Non-participant observation was conducted in three livestock markets, three zoo-sanitary checkpoints, 20 slaughter facilities and 15 dip sites.

**Table 2:** Demographic characteristics of the survey respondents.

**Characteristics**		**Number of respondents (*n*)**
Gender	Male	26
	Female	7
Age	21–30	1
	31–40	17
	41–50	10
	51–60	5
Education level	Certificate	2
	Diploma	26
	Bachelor degree	2
	Higher degrees	3
Work experience	1–5 years	4
	6–10 years	18
	>10 years	11

### Implementation of Animal Health Surveillance in Tanzania

#### Fidelity

Standard operating procedures require every suspected case to be confirmed in the laboratory. However, it was reported that it was reported that case identification was mainly based on clinical signs and symptoms due to limited access to laboratory services. Sample collection for laboratory examination was reported to be very rare due to limited and distant diagnostic facilities. Although data collection and reporting were supposed to be done through designated surveillance forms, only 27% of the respondents (*n* = 33) reported using them while the rest maintained their records on notebooks. Notification of the disease events was mostly verbal using mobile phones or short message services with limited formal documentation. There were multiple reporting channels, including the Agricultural routine data system (ARDS), disease surveillance form, and Event mobile application (EMA-i), but they were not coordinated causing a risk of double-counting the event. Eighty four percentage of LFOs (*n* = 30) reported to physically transmit surveillance reports to the district offices and transport costs were covered out-of-pocket. Data transmissions from the districts to ZVCs and MoLF were done via emails, but the internet expenses were covered by the sender.

#### Adherence

The study found that there were different reporting levels and timelines. At the district levels, LFOs submitted a brief report on the disease status every Thursday via SMS, and monthly ARDS reports had to reach the district desk by the 5th day of the new month. The weekly reports from the districts were forwarded to ZVCs and ultimately reached the ministry desk by Monday. The record review revealed that most of the collected data by the LFOs were filled in the ARDS and submitted to the Ministry of Agriculture through local government authorities (LGAs), but that information never reached the MoLF for analysis because there was no direct communication link between the two ministries on surveillance related matters and the reporting systems were not linked.

**Figure 5:**
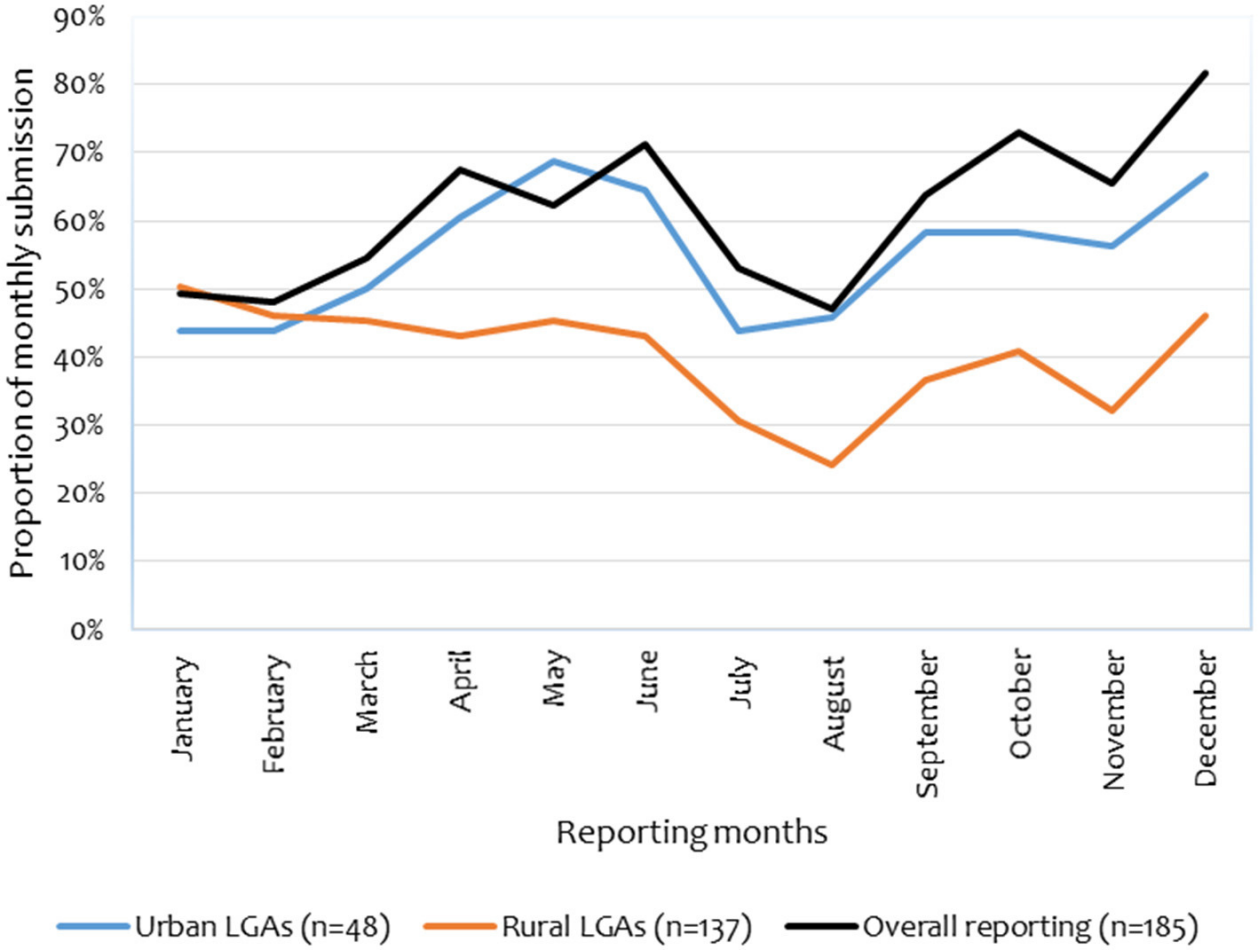
Proportion of monthly reports received by the MoLF from LGAs throughout the year.

At the national level, the proportion of monthly reports submitted from the LGAs for the year 2020 was 61% (number of LGA, *n* = 185) (range 47–82%). It was also found that 99% of the reported events were from the rural LGAs (refers as DCs), but proportionally, their average reporting annually is lower (40%, *n* = 137) as compared to the urban LGAs (55%, *n* = 48) which include city councils, municipalities and townships. Figure 5 depicts a lack of consistency in reporting, although it is mandatory. The sharp rise on the curve from September was explained by the intervention from veterinary council of Tanzania (VCT), which directed all the DVOs to send the report as required by the law; otherwise, necessary action would be taken against them.

#### Exposure

**Figure 6:**
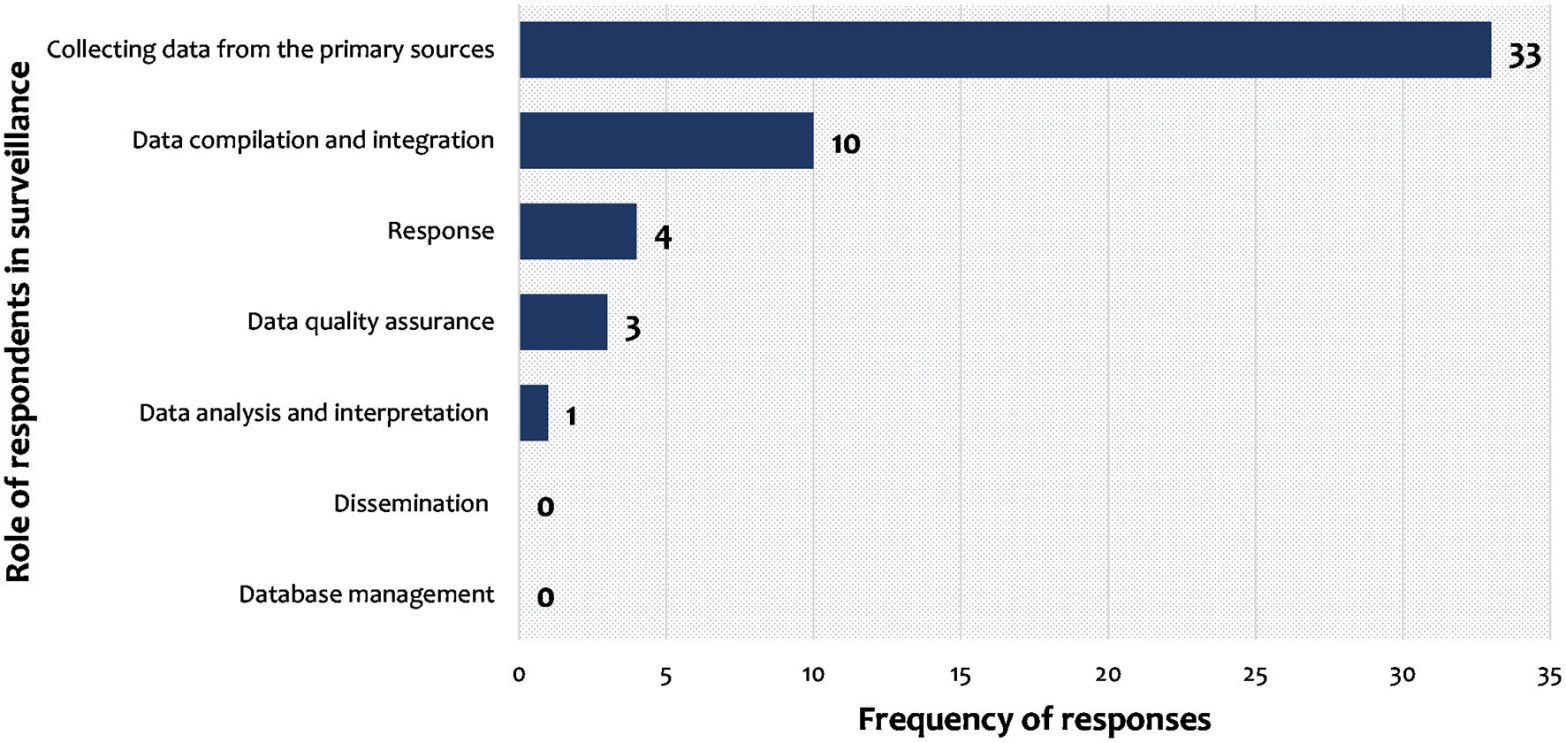
Role played by respondents in surveillance activities.

Figure 6 shows the level of which respondents were engaged in animal surveillance activities. They were mainly engaged in mainly in data collection from the primary sources (33/33), and data compilation and integration (10/33). However, they rarely received feedback on the data they sent, they reported that to be demotivating. Only 30% of the LFOs (*n* = 30) have ever received any training related to surveillance, while DVOs and other officers at higher levels reported having received regular trainings on the same.

### Mechanisms of Impact

#### Satisfaction

**Figure 7:**
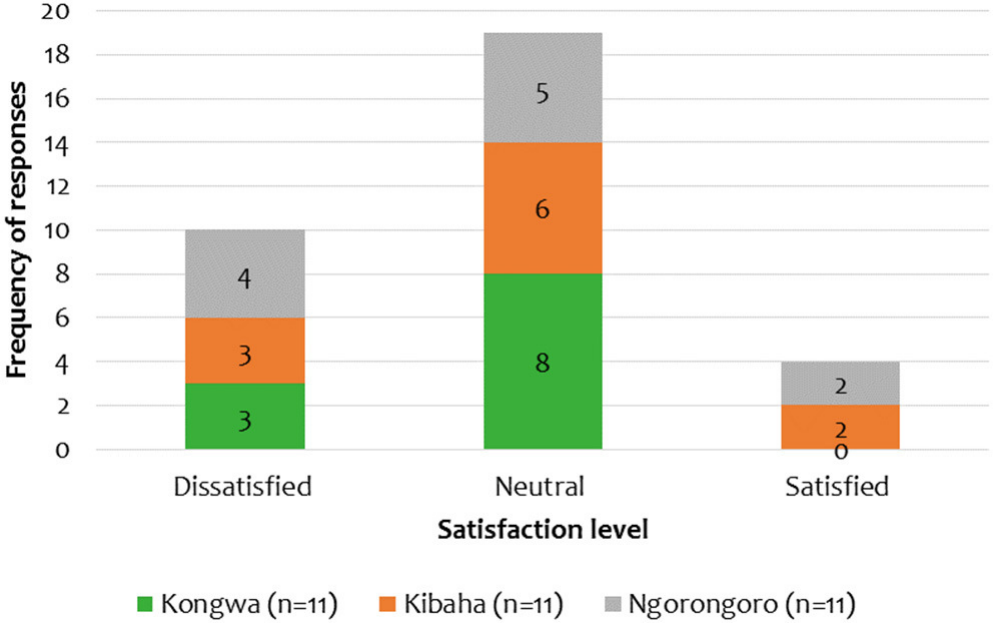
Satisfaction level of the respondents to the current surveillance system.

Figure 7 depicts the satisfaction level of respondents in each study district. When asked to rank their satisfaction with the current animal surveillance system on a scale of 1–5 (1 = very dissatisfied, 2 = dissatisfied, 3 = neutral, 4 = satisfied, 5 = very satisfied) but during analysis were collapsed into scales 1–3 (1 = satisfied, 2 = Neutral, 3 = dissatisfied). Nineteen out of thirty three (58%) respondents were neutral on their satisfaction with the surveillance system, while only 4/33 (12%) were satisfied. Some of their dissatisfaction was explained to be caused by high transport costs, hard working conditions, lack of feedback and limited human resources.

#### Participation of LFOs in Surveillance Activities

The majority of the LFOs (20/30) use 20–90% (mean = 61.2%, SD = 20.4%) of their time per week for surveillance-related activities, while DVOs spent an average of 60% on the same. The level of effort varied among the districts; LFOs in Kibaha dedicated more time to surveillance activities than those from Kongwa and Ngorongoro (Table 3). Field-level staff reported to be overwhelmed by other responsibilities which were not animal health-related, including administration roles and revenue collection.

**Table 3:** Proportion of time district level animal health officials spent on surveillance activities.

**District**	**Proportion of time spent in surveillance activities (in percentage)**				
	**<20 (%)**	**20–40 (%)**	**41–60 (%)**	**61–80 (%)**	**>80 (%)**
Kongwa (*n* = 10)	0	40	20	40	0
Kibaha (*n* = 10)	0	0	30	60	10
Ngorongoro (*n* = 10)	0	20	40	20	20

#### Recruitment, Trainings and Technical Assistance

All LFOs in Kibaha and Ngorongoro have a diploma or degree in animal health sciences, but in Kongwa, only three of the 22 extension officers have an animal health background, the rest have a diploma or certificate in general agriculture. Officials from the district to the ministerial level have bachelor degrees in veterinary medicine or animal health sciences. All respondents were employed at entry level in their respective wards and have served with 4–29 years in their current positions (mean = 12, SD = 7.7 years), but only 33% (*n* = 30) and 30% (*n* = 30) of them have ever received on-job and surveillance trainings, respectively. The last training was 5 years ago (min = 1, max = 15). The trainings were mainly about artificial insemination, Tsetse control, vaccination or animal health management. Field supervisions were irregular due to financial constraints.

### Contextual Factors Affecting the Implementation of Animal Health Surveillance

#### Area Coverage per Designated Officer

Average area coverage per ward LFO was 5 villages (Kongwa = 6, Kibaha = 4, Ngorogoro = 4). During the implementation of activities, LFOs traveled long distances to serve their livestock keepers. In Kibaha, LFOs traveled an average of 192 kms, Kongwa 209 kms and 328 kms every month for animal health-related activities, including surveillance. Either livestock keepers or LFOs themselves bore the travel costs all the time. In providing veterinary services and implementing surveillance activities, wards were supervised DVOs or DLFOs while LGAs were under respective ZVCs. However, the supervisors have to travel long distances to reach some wards and LGAs, as indicated in Table 4.

**Table 4:** Area covered and furthest distance traveled by the DVOs and ZVC officers.

**ZVC**	**Number of LGAs**	**Farthest LGAs (in kms)**	**Selected LGAs**	**Number of wards**	**Farthest ward (in kms)**
Eastern	22	560	Kibaha DC	22	87
Central	15	350	Kongwa	14	100
Northern	32	550	Ngorongoro	28	320

#### Field Level Human Resource vs. Livestock Population

In the three studied district councils, the common livestock kept were cattle, goat and sheep. There were 828,904 cattle, 886,044 goats and 889,978 sheep as of 2019. The availability of the ward LFOs with animal health backgrounds was only 50% of what was required (Table 5) with kongwa being the most constrained (3/22) in animal health professionals. However, Ngorongoro had a high number of livestock to LFO ratio because it had the highest number of livestock. To reduce the gap, in some areas, ward agricultural extension officers were being used to provide livestock extension services and other LFO responsibilities. LFOs reported to be serving multiple roles in the field, including providing livestock extension services to livestock keepers, collecting surveillance data, delivering meat inspection in slaughter facilities, and collecting revenue in livestock markets. In some places, they were found to assume administrative roles such as acting as ward executive officers (WEOs).

**Table 5:** Field level human resources vs. livestock population in the selected district councils in 2019.

**District council**	**Cattle**	**Goats**	**Sheep**	**Total**	**Available LFOs**	**Required**	**No. of Livestock/LFO**
Kongwa	121,973	79,793	36,662	238,428	3	22	79,476
Kibaha	51,408	18,379	7,121	76,908	14	14	5,493
Ngorongoro	655,523	787,872	846,195	2,289,590	15	28	152,639
Total	828,904	886,044	889,978	2,604,926	32	64	81,404

*Source: District veterinary officers*.

#### Costs of Surveillance Data From Field to the Epidemiology Unit

**Figure 8:**
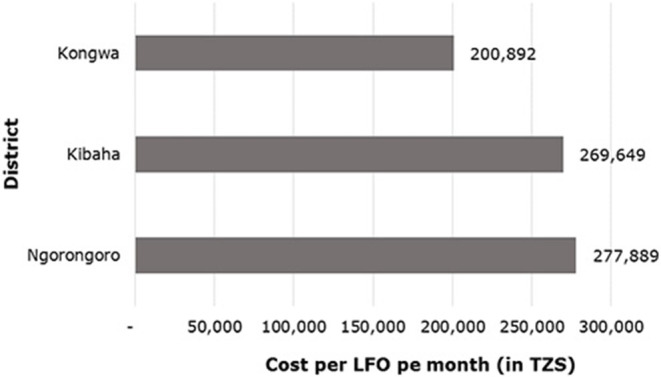
Monthly cost of surveillance data incurred by each LFO in Tanzanian shillings (TZS).

The annual cost of surveillance data national-wide was 12,168,085,833 TZS. Field level data collection and transmission accounted for 97% of the total cost (11,760,238,560 TZS). Cost of surveillance per ward LFO ranged from 80,740 to 828,200 TZS per month (mean = 247,730 TZS). Ngorongoro had the highest total surveillance costs per LFO compared to other districts (Figure 8). When asked whether they received transport allowance for those who transmit data physically, 83% of respondents (*n* = 30) reported to use their own funds without re-imbursement by the employer. Only two respondents who were working in the Ngorongoro Conservation Authority Area (NCAA) reported to receive facilitation allowance.

#### Communication and Supporting System

The DVOs or DLFO were the primary recipients of surveillance data, except for NCAA, where LFOs sent reports to NCAA-Veterinary officer who then forwarded them to the DVO. Apart from sending reports to the district, 40% of the ward LFOs (*n* = 30) reported sharing the reports with the ward development council. There was no direct communication with LFOs beyond the district level. Inter-sectoral collaboration, especially between animal health sectors professionals and other sectors, was strong at the field level, as reported by 70% of the LFOs who mainly collaborate with human health professionals. Kibaha had the strongest collaboration (90%) while Kongwa had the weakest (50%). Refresher training on surveillance for the field level staff was very low as only 27% LFOs reported to have received training. In most cases, they used the college and field experience knowledge to solve day-to-day animal health-related challenges.

Commonly reported challenges at the field level were lack of transport (21/33), no facilitation for the collection and transmission of surveillance data (17/33), low awareness of livestock keepers on the importance of disease reporting (14/33), lack of working equipment and tools such as sample collection kits and data collection tools (13/33), and limited workforce compare to the vastness of the areas (12/33). Other reported challenges were the low response rate from higher authorities on the reported cases, poor veterinary infrastructures, lack of a coordinated filing system leading to the reports getting lost, political interference and a limited laboratory network for sample confirmation.

#### Legislation and Reporting Obligations

Animal health surveillance in Tanzania is governed by national and international guidelines and legal frameworks. First, surveillance is guided by the OIE Terrestrial animal health code, which sets standards for improving the health and welfare of terrestrial animals and veterinary public health worldwide. Second, at the national level, there were several policy and legal frameworks guiding animal health surveillance and disease control which spelt out processes and roles and responsibilities of various players as indicated in Table 6.

**Table 6:** Legal documents guiding animal health surveillance in Tanzania.

**Legal document**	**Year**	**Provision**
National Livestock Development Policy	2006	The Government to strengthen technical support services in animal health, control and eradication of Trans-boundary animal diseases, tick-borne, tsetse flies and trypanosomes control and other diseases of economic importance
Animal Disease Act, No. 17 of 2003	2003	Mandate of DVS in disease prevention and control, powers of inspectors, compulsory measures and general provisions for disease prevention and control
Veterinary Act No 16 of 2003	2003	Registration of veterinarian, paraprofessionals and paraprofessional assistants and retention requirements and registration of veterinary practice facilities
Local Government (District Authorities) Act of 1982	1982	Regulate livestock movement
Livestock Registration, Identification and Traceability Act No 12 of 2010	2010	Spelled out the purpose of the act, which among others was controlling animal diseases
Animal Diseases Regulations	2005	Power and duties of inspectors in disease identification, prevention and control

However, the execution of the mandate as stipulated in the legal frameworks was still a challenge, especially at the field level. Some of the difficulties reported were: interference of local government administration in the quarantine exercises especially when it would compromise the revenue collection; a limited number of animal health officers vs. the vastness of the designated areas, limited budget allocation and political influence on surveillance and animal health activities, e.g., registration of animal into Tanzania livestock identification and traceability system as per law. The Veterinary Act 2003 spelt out eligibility for registration of animal health practitioners. 76.7% of the respondents (*n* = 33) were eligible and registered as professional or paraprofessional assistants.

#### Financial Resource Allocation vs. the Cost of Surveillance Data

Since DVS was responsible for disease prevention and control, including surveillance, every year, the budget had been allocated to implement such activities. However, a 5-year budget analysis (2015/2016–2019/2020) showed that DVS budget allocation had been consistently low, with an average of 8,832,835,768 TZS per year (21% of the total ministerial budget). The budget entailed personal emolument, development and other charges. It was allocated to facilitate veterinary laboratory services, implement disease control strategies, strengthen epidemiological surveillance, and strengthen ZVCs, among other budget items. On average, 32% (2,870,630,149 TZS) of the total DVS budget was directed exclusively to surveillance and disease control.

**Figure 9:**
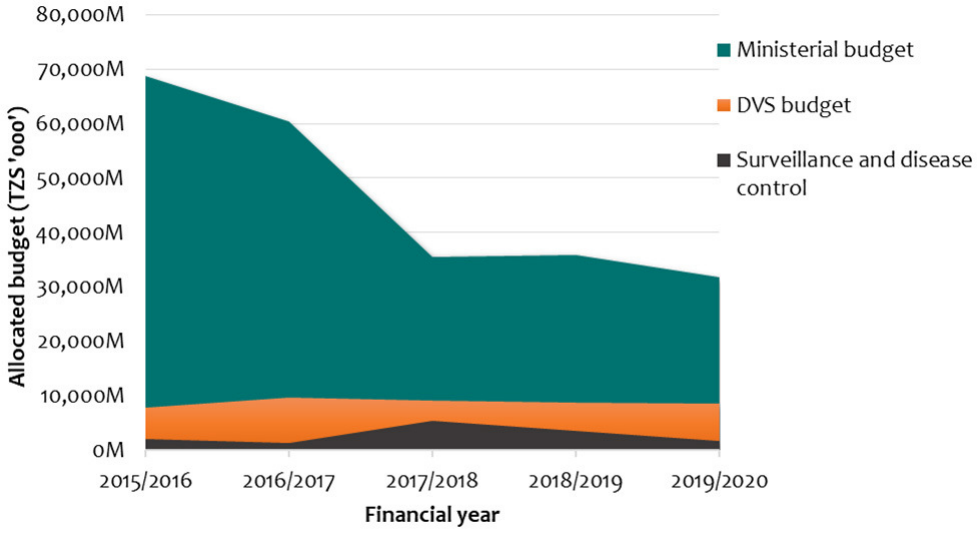
Budget allocation for surveillance and disease control against DVS and ministerial budgets.

Figure 9 illustrates the trend on DVS budget allocation to surveillance and disease control in comparison to the ministerial budget over 5 years. The sharp rise in the 2017/2018 financial year for surveillance and disease control was accounted for by the budget allocated to TVLA for developing the vaccine institute. On the other hand, the cost of surveillance data was approximated at 12,168,085,833 TZS per year, which is 1.4 times higher than the annual national surveillance and disease control budget.

### Linkage Between Surveillance Processes and System Attributes

#### Data Quality

There were guidelines for surveillance, including animal diseases surveillance field reports and veterinary services abattoir report forms, but they were rarely used. It was reported and observed that LFOs recorded their activities on the notebooks. Weekly reports on the events were sent through short message service (SMS) or social media group (WhatsApp) to DVOs/DLFOs outlining what has happened in that particular week. The excel database at the epidemiology unit showed the inconsistency in reporting, and data in the sheets were not the reflection of the information on the surveillance forms. The review of the dataset identified the following weaknesses potentially affecting the data quality: (i) lack of consistency on data, e.g., a data entry can have different labels for the same item, such as Dodoma, Dodoma MC, Dodoma council, Dodoma city council, Dodoma CC or Chemba and Chemba DC or for the dates 24–29/January 2020 vs. 30 JANUARY-05 FEBRUARY 2020, 31st January to 6 February 2020, (ii) incomplete data, (iii) data were not in analyzable format, (iv) and (v) typing errors.

#### Simplicity

It was reported several sources generated surveillance data, including livestock farmers, veterinary facilities such as markets and slaughterhouses/slabs and zoo-sanitary checkpoints. It was also observed that there were multiple reporting channels such as ARDS, animal disease surveillance form, abattoir forms, phone calls, and SMS reporting similar information; however, they were not synchronized thereby creating a risk of double counting certain events. Data were compiled and cross-checked at district and zonal level Overall analysis and reporting were done at the ministry level from weekly and monthly reports from ZVCs. Every 6 months, data cleaning and analysis are done to prepare semi-annual reports. However, data extraction and cleaning reported and observed to be tedious process for the data analysts and takes long due to lack of standardized formats. At the ministry level, surveillance reports were submitted to different users, including international bodies such as OIE, SADC, AU, and EA, with varying reporting formats. Feedback to the field staff was reported to be limited.

#### Timeliness

Weekly reports were to be submitted by LFOs to the DVO by Wednesday of every week to reach the ministry desk by Monday. A total of 2,255 reported disease events were extracted from EMPRES-I, whereby 95.6% of them are from rural LGAs. The median reporting delay ranged from 0 to 153 days from the observation date (median = 2 days, mean = 6 days).

#### Usefulness

Surveillance data were mainly used at the national level to design interventions such as rabies, anthrax, or Peste des Petits Ruminants (PPR) control, to prepare the list of priority zoonotic diseases, to report to international bodies, and to rollout of vaccination programmes throughout the country for the selected list of diseases. Although other stakeholders such as traders were able to access data upon enquiry, they rarely made such requests.

## Discussion

The objective of the study was to evaluate surveillance processes and the contextual factors which facilitate or hinder uptake, implementation and sustainability of the system. The study indicated that there were clear reporting structures, guidelines and legal frameworks for implementation of animal health surveillance in the country. Most of the field-level staff had the desired qualifications and there was strong intersectoral relationship. Nevertheless, there were deviations from standard processes and procedures in some of the aspects of the surveillance such as limited coordination of data collection with multiple reporting channels and lack of refresher trainings for the staff. That has led to low adherence including low monthly submission and poor quality of data. The cost of surveillance data was found to be 1.4 times higher than the annual surveillance budget. Generally, the study revealed the interconnectedness between the animal health surveillance processes, contextual factors and outcomes which may need holistic analysis. Findings from this study may help provide more insights on its implementation.

Implementation of the surveillance activities was assessed in terms of fidelity, completeness and exposure to the system. It was found that there were deviations from standard guidelines, especially in case detection and reporting. Case detection, the critical component of any surveillance system, was primarily through visual observations due to limited diagnostic facilities. Although the laboratory system is an important pillar of surveillance, Tanzania's network of laboratories has only eleven units (24), and laboratory test results were not well-mainstreamed into the surveillance system (16). Laboratory tests can contribute to early warning by analyzing the test requests and diagnosis (25), but very few suspected cases reach the laboratories (26). Effective animal health surveillance systems require reliable, high-quality, and timely data for decision making. Designated disease surveillance forms were not being used by majority of the LFOs because of difficult in accessing them especially where they don't have access to stationary services hence they opted to send by SMS or phone calls which also have limitations. The evaluation found high reliance of the system on data from livestock keepers and slaughter facilities mostly collected by LFOs during their routine animal health activities. At the same time, other sources such as dipping sites and veterinary shops were not being utilized. The reporting system was fragmented with multiple reporting channels, which were not unified hence lead to the loss of some of the data on the way. George et al. (16) demonstrated how available data sources could be leveraged to improve Tanzania's animal health surveillance system by banking on their complementary strengths.

Tanzania's animal health surveillance is mostly passive, considered the most cost-effective approach for early warning and outbreak detection. However, its performance depends on the users' acceptability, attitude, participation, and understanding of how the system works. It was noted that despite the high participation of subnational level officials in surveillance-related activities, they received little to no training on the surveillance. Several studies emphasized the importance of frontline workers in increasing the ability of the system to detect and contain infectious diseases (26–28). The recruitment of designated surveillance officers must be in line with national and international competency guidelines (29–31). More than 76% of animal health officials in the field qualified to be surveillance officers, but only 30% of them had ever received training on animal health surveillance. This could be contributing to the poor quality of submitted data and low compliance with surveillance protocols. Improving the performance of the frontline workers in surveillance requires changes in capacity building and management particularly motivation mechanisms, supervision system, clear communication and regular refresher trainings to keep up with the demand for quality and timely data.

The usefulness of an early warning surveillance system lies in its ability to detect any anomaly timely, and that information is used to decide on disease prevention or control. Despite the limitations identified, Tanzania's surveillance system was reported to be useful in designing interventions. Nonetheless, the time lapse between the occurrence of the event and reporting compromises the sensitivity of the system to detect outbreaks timely. This attribute is most needed in this era of a high rate of new emerging and re-emerging infectious diseases. The findings were consistent with the 2020 performance audit report (32). Some of the contextual factors that accounted for those setbacks included the vastness of the areas that are not proportional to the available workforce, multiple and sometimes conflicting roles of the LFOs, poor communication, the surveillance organizational structure, and the high cost of surveillance data. To address these challenges and enhance passive surveillance, there is a need for public awareness and education on the importance of timely reporting and incentivization of reporting, especially on unusual cases (33). The data collection process should be easy and integrated into routine works of the subnational level animal health officials to maintain their participation. The system may also benefit from integrating available data sources while leveraging digital tools such as AfyaData and EMA-I to reduce transmission costs and improve data quality.

In most of the low-income countries, animal health surveillance budgets are heavily dependent on public and donor funding. Still, for a long time, there has been a funding gap in the resources required to carry out surveillance for disease control as compared to human-health related surveillance (34). Tanzania's budget allocation has also demonstrated the same trend in the past 5 years with no clear pattern. The analysis also revealed that a large portion of the budget went into staff costs compared to the surveillance and disease control activities. This affects the performance of surveillance systems and disease prevention and control in general. The main methodological challenges in the budget analysis have always been the inaccessibility of quality data during data collection and extraction (35, 36). For instance, in this study, the researcher resorted to using only allocated budget instead of actual expenditures because data were fragmented, not straightforward, and others were missing. However, this situation is expected to be reversed soon as the country moving toward e-Government services, including the operationalization of the Government Accounting System (MUSE). Despite having the third-largest livestock population in Africa, the livestock sector contribution to the gross domestic product (GDP) is only 7.4 percent and growing at 2.2 percent annually (37). Therefore, to challenge the status quo, the government has to take deliberate efforts to invest in surveillance to prevent avoidable disease outbreaks, which come with high economic impact. It should explore financing mechanisms leveraging on the public-private partnerships.

Institutional arrangement and collaboration between government institutions and other stakeholders play a significant role in influencing the implementation of surveillance activities. Inter-sectoral collaboration between animal health professionals and other stakeholders at the subnational level was commendable. However, a lack of coordination and communication was observed between the MoLF and PO-RALG, the custodian of all LGAs on animal health surveillance, especially reporting. Tanzania Public Service Act, Cap 298 R.E 2019 and The Local Government Laws (Miscellaneous Amendments) Act, 2006 spelt out the mandates of LGA and sector ministries to local government public servants, but its execution has always been challenging due to unclear chain of commands (32). In order to rectify that, the two ministries may have a memorandum of understanding that all matters related to surveillance and animal health should be accounted directly to the sectoral ministry. Alternatively, all the staff working in animal health should be answerable to the sectoral ministry for easier communication and greater accountability. Institutional collaboration is increasingly necessary because some of the interconnected challenges can only be tackled interorganisationally (38). The growing demand for the operationalization of One Health also emphasizes the importance of improving communication between ministries, especially for zoonotic diseases.

The process evaluation was for the national surveillance system and data were collected at all levels. Due to limited resources, authors selected three districts as case study for the district-level representatives which may not be representation of the whole country but complement information collected at other levels. In the analysis of financial resources for animal health surveillance, authors opted to use budget allocates instead of actual expenditures due to inaccessibility of data. This may have led to overestimation of the funds spent on surveillance activities but may suffice in providing insights on financial resource allocation.

## Conclusion

This study assessed the implementation of the animal health surveillance system in Tanzania and contextual factors that affect the system performance. The study revealed that the uptake on the recommendations from the previous evaluations was low; therefore, there were deviations in implementing surveillance from its core principles and standards that affected the system's performance, especially on data quality and timeliness, necessary for early warning. The study also showed barriers to the functionality of the surveillance system, including a large area coverage, lack of funding, limited law enforcement, challenges in communication and supporting systems and high cost of surveillance data. Therefore, this process evaluation showed that for the animal health surveillance to improve, it requires the integrated and coordinated mechanisms that are sustainably funded. User-friendly unified reporting system, active involvement of subnational level animal health officials and optimization of available data sources while tapping into digital tools may reduce the cost of data and improve timeliness and data quality. Finally, the government should explore other financing mechanisms besides from the national budgets, such as re-investing certain percent of its collected revenue into disease control and surveillance and leveraging the public-private partnership.

## Data Availability Statement

The original contributions presented in the study are included in the article/supplementary material, further inquiries can be directed to the corresponding author/s.

## Ethics Statement

This study was approved by Research Ethics Committee of Sokoine University of Agriculture. It was granted research clearance from the Vice Chancellor on behalf of Tanzania Commission for Science and Technology (COSTECH), reference no. SUA/ADM/R.1/8/331-335. Participants gave written consent after reading consent declaration form.

## Author Contributions

JG conceptualized the study with critical inputs from BH, EK, JM, MR, and SK. JG collected, analyzed and interpreted data, and wrote the first draft of the manuscript. BH, EK, JM, MR, and SK provided oversight throughout data collection, analysis and interpretation of the results, and substantively revised the first draft of the manuscript and subsequent modifications. All authors contributed to the article and approved the submitted version.

## Funding

The study was part of research on the development of a prototype for cost-effective integration of animal health surveillance systems in Tanzania supported by the Government of Tanzania and World Bank, grant no. PAD 1436 through SACIDS Africa Centre of Excellence for Infectious Diseases of Humans and Animals in Southern and Eastern Africa (SACIDS-ACE). The funder had no role in the design of the study, data collection, analysis and interpretation or preparation of this manuscript.

## Conflict of Interest

The authors declare that the research was conducted in the absence of any commercial or financial relationships that could be construed as a potential conflict of interest.

## Publisher's Note

All claims expressed in this article are solely those of the authors and do not necessarily represent those of their affiliated organizations, or those of the publisher, the editors and the reviewers. Any product that may be evaluated in this article, or claim that may be made by its manufacturer, is not guaranteed or endorsed by the publisher.
